# Whole-Genome Sequencing of Lactobacillus johnsonii MT4, a Novel Strain Isolated from the Oral Cavity of C57BL/6 Mice

**DOI:** 10.1128/mra.00089-23

**Published:** 2023-04-12

**Authors:** Roberto Vazquez-Munoz, Jordan T. Russell, Angela Thompson, Yanjiao Zhou, Anna Dongari-Bagtzoglou

**Affiliations:** a Department of General Dentistry, University of Connecticut Health Center, Farmington, Connecticut, USA; b Department of Medicine, University of Connecticut Health Center, Farmington, Connecticut, USA; University of Rochester School of Medicine and Dentistry

## Abstract

Lactobacillus johnsonii strain MT4, isolated from the oral cavity of C57BL/6 mice, elicits antimicrobial activity against disease-associated microorganisms. Short-read sequencing of the whole genome revealed a single genome of 1,883,026 bp, with a GC content of 34.4%, and no plasmids.

## ANNOUNCEMENT

Lactobacillus johnsonii is associated with probiotic properties, host immunomodulation, and mitigation of certain metabolic syndromes (1–3). MT4 displays antibacterial and antifungal properties (4, 5).

MT4 was initially isolated by our group from C57BL/6 mice tongues and identified as L. johnsonii, as described by Bertolini et al. (4). Pure cultures were incubated overnight in de Man, Rogosa, and Sharpe broth under static, anaerobic conditions at 37°C. Cells were lysed using a custom lysis buffer (6) and zirconia beads. Total DNA was extracted using the DNeasy blood and tissue purification kit (Qiagen).

Purified genomic DNA (gDNA) was quantified using the double-stranded DNA (dsDNA) high-sensitivity assay for Qubit 3.0 (Life Technologies, USA). gDNA fragmentation was analyzed on the Agilent 4200 TapeStation using the genomic DNA assay (Agilent Technologies). gDNA (1 ng) was normalized to 0.2 ng/μL for whole-genome shotgun library preparation using the Illumina Nextera XT library preparation kit (Illumina), according to the manufacturer’s instructions. The libraries were validated for length (average length, 450 bp; average insert size, 315 bp) and adapter dimer removal using the high-sensitivity D5000 ScreenTape assay (Agilent Technologies). The libraries were then quantified and normalized using the dsDNA high-sensitivity assay for Qubit 3.0 and prepared for Illumina sequencing by denaturing and dilution. The sample was run on the Illumina NovaSeq 6000 SP 300-cycle sequencing kit with v1.5 chemistry. The target read depth was achieved with paired-end (PE), 150-bp reads. The sequencing reads were filtered based on Illumina base-calling software algorithms. Quality control was assessed by analyzing forward and reverse FASTQ sequences in the Computational Biology Core High Performance Computing (HPC) facility and phiX reads were filtered from both paired-end FASTQ files using the “filter_phix” command with default parameters in USEARCH v10.0.240 (7). FASTQC was used to assess the sequence data quality and adapter content. The sequence data were high quality (>Q30 throughout) and did not require additional quality filtering. Approximately 10% of paired-end reads contained Nextera adapter sequences, which were trimmed using Trimmomatic v0.39 in PE mode using the provided Nextera adapter file and keeping a minimum trimmed read length of >36 bases (8).

In total, 3,026,429 read pairs were obtained, of which 278,192 forward and 278,217 reverse reads were trimmed to remove Nextera sequences. The trimmed reads were assembled *de novo* using SPAdes v3.15.2 (9) with default parameters through Unicycler v0.4.8 to assemble the genome from both paired-end FASTQ files. The final assembly consisted of 68 contigs (*N*_50_, 90.96 kb) and had a total length of 1,883,026 bp, with a GC content of 34.4%, in a single genome with no plasmids. The genome assembly averaged a sequencing depth of 240×. The MT4 assembled genome was annotated using Prokka v1.14.6 (10), followed by PGAP, after submission to the NCBI GenBank database. Using PGAP, 1,826 genes were predicted, including 1,740 protein-coding genes, 60 RNA genes (3 noncoding RNAs [ncRNAs], 3 rRNAs, and 54 tRNAs), and 26 pseudogenes. The MT4 genes include antimicrobial-associated bacillomycin D, surfactin, and MspI/p75 (5).

Genome-based taxonomy, using Kraken2 v2.0.8-beta (11), confirmed MT4 as an L. johnsonii strain (average nucleotide identity, 98.53%). A phylogenetic analysis on the core genes of 17 L. johnsonii strains found at NCBI (5) using Roary v3.13.0 (12) revealed that MT4 is closely related to the probiotic strain NCC533 (La1). Roary was run with default parameters, specifying core gene alignment performed using PRANK, core gene presence in 99% of included strains, and 95% blastp protein identity.

### Data availability.

The assembled MT4 genome sequence and related data can be found under GenBank accession number JAJQJG000000000, BioProject accession number PRJNA787656, and BioSample accession number SAMN23838460. The raw FASTQ sequencing data are available under SRA accession number SRR17309641.
